# Hyperpolarization-activated cyclic nucleotide-gated channels and epilepsy: genetics, circuits, and treatments

**DOI:** 10.3389/fneur.2025.1744653

**Published:** 2026-01-12

**Authors:** Ming-Shan Cai, Peng Liao, Zhen-Liang Hu, Yun Li, Jia-Xing Zhao, Liang Jin, Hong-Wei Wang, Yong-Jun Chen

**Affiliations:** 1 Department of Neurology, The Affiliated Nanhua Hospital, Hengyang Medical School, University of South China, Hengyang, China; 2 Department of Brain Function and Neuroelectrophysiology, The Affiliated Nanhua Hospital, Hengyang Medical School, University of South China, Hengyang, China; 3 Department of Neurology, The First Affiliated Hospital, Hengyang Medical School, University of South China, Hengyang, China

**Keywords:** circuits, epilepsy, genetic mechanisms, HCN channels, targeted therapies

## Abstract

Epilepsy is a common chronic disorder of the central nervous system characterized by recurrent seizures arising from abnormal, hypersynchronous neuronal activity; its pathogenesis is complex and remains incompletely understood. Hyperpolarization-activated cyclic nucleotide-gated (HCN) channels are a family of voltage-gated ion channels that mediate the hyperpolarization-activated cation current (I_h_) and play key roles in regulating neuronal excitability, rhythmic activity, and synaptic transmission. Recent studies indicate that abnormal HCN channel expression, pathogenic genetic variants, or dysregulated protein interactions are closely linked to the onset and progression of epilepsy and may contribute to disease by destabilizing membrane-potential homeostasis, perturbing neurotransmitter balance, and disrupting network-level control of excitability. This review summarizes the structural and functional properties of HCN channels and focuses on their mechanistic roles in epileptogenesis, with the goal of informing clinical diagnosis and therapeutic development.

## 1 Introduction

Epilepsy is one of the most common neurological disorders worldwide, affecting approximately 50 million people, with a disproportionate burden in low- and middle-income countries (1). Despite antiseizure medications (ASMs) being the mainstay of therapy, about one-third of patients continue to have seizures and meet criteria for drug-resistant epilepsy (DRE) (2, 3). Beyond recurrent seizures, epilepsy is closely associated with cognitive impairment, reduced quality of life, and limitations in social participation (4). Etiologies are heterogeneous and, under the ILAE framework, include structural, genetic, infectious, metabolic, immune, and unknown causes (5). The contribution of genetics is increasingly recognized across epilepsy types: large-scale exome studies and recent reviews show that rare/ultra-rare and common variants jointly contribute to risk, with significant enrichment in pathways governing synaptic transmission and neuronal excitability (6–8). Among these, ion channels—core regulators of electrical signaling—occupy a central position; multiple channelopathies have been shown to directly cause epilepsy or profoundly influence seizure threshold and treatment response (7–9).

Among ion channels, HCN channels—by virtue of their unique hyperpolarization-activated gating and precise regulation of neuronal rhythmicity and excitability—have become a major research focus in epilepsy (10–12). HCN channels were first identified in cardiac sinoatrial nodal cells (13), where they mediate the “pacemaker current”—a current originally named for its role in generating intrinsic rhythmic activity that sustains cardiac rhythm (14, 15). Notably, while the “pacemaker” terminology originates from cardiac physiology, HCN channels in the central nervous system (CNS) do not typically serve a direct pacemaker function; instead, they primarily regulate neuronal rhythmicity and synchronization, which are critical for maintaining network stability. Subsequent studies demonstrated their broad distribution throughout the CNS, with particularly high expression in seizure-relevant regions such as the hippocampus, cerebral cortex, and thalamus (16–18). By carrying the hyperpolarization-activated cation current (I_h_), HCN channels regulate the resting membrane potential, action potential firing rate, and presynaptic neurotransmitter release, thereby directly shaping neuronal excitability and network stability (19, 20).

## 2 Structure and functional properties of HCN channels

### 2.1 Molecular architecture and tissue distribution

**Figure 1:**
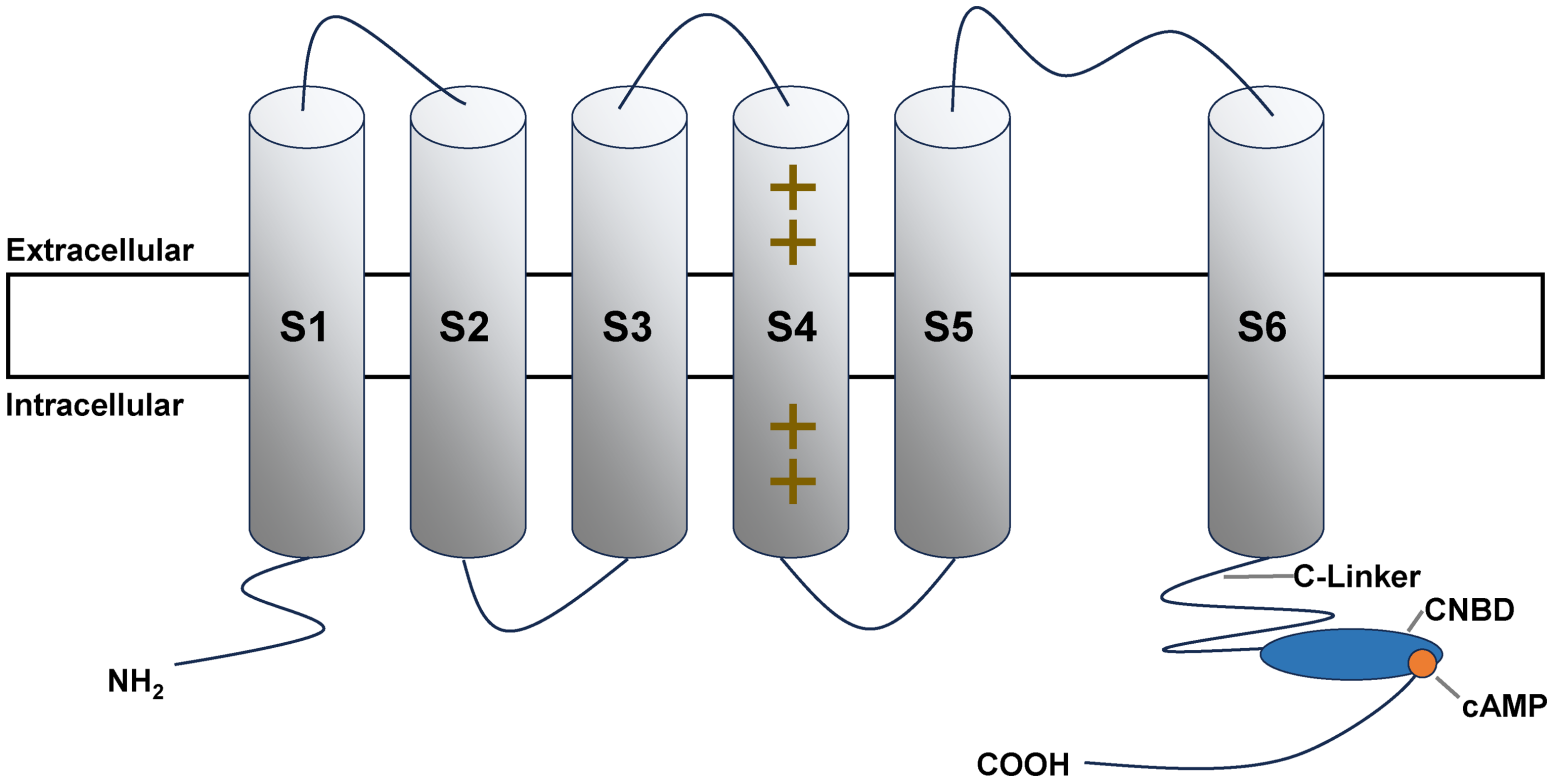
HCN channel subunit. It comprises six transmembrane segments (S1–S6). The S4 segment contains positively charged residues, serving as the voltage-sensing domain. At the proximal carboxyl (COOH) terminus, a C-Linker links to the cyclic nucleotide-binding domain (CNBD), which interacts with cyclic adenosine monophosphate (cAMP) to mediate channel regulation. The extracellular and intracellular compartments are indicated. NH₂ represents the amino terminus.

HCN channels are specialized members of the voltage-gated ion-channel superfamily (Figure 1). Each functional channel is a tetramer of homologous subunits; every subunit contains six transmembrane segments (S1–S6) (21), a pore-forming region between S5 and S6 that confers ion selectivity (22, 23), and an intracellular C-terminal cyclic nucleotide-binding domain (CNBD) of ~120 amino acids (24, 25). The CNBD is a key regulatory module that binds cyclic nucleotides—cyclic adenosine monophosphate (cAMP) or cyclic guanosine monophosphate (cGMP)—and modulates channel gating via conformational coupling (21, 23, 26–29). Upon cAMP binding, channel open probability increases and the activation curve of the I_h_ current shifts toward more depolarized potentials, thereby enhancing channel responsiveness to hyperpolarizing stimuli (27, 30).

At the protein level, the mammalian HCN channel family comprises four isoforms (HCN1-HCN4) (22). Although these isoforms share ~60–80% amino-acid identity, they differ substantially in regional distribution, biophysical properties, and modes of regulation (25). In the CNS, HCN1 and HCN2 are the predominant isoforms: HCN1 is widely expressed in neocortex, hippocampus (especially CA1 pyramidal neurons and dentate granule cells), cerebellar Purkinje cells, and thalamic neurons; it activates most rapidly and is the least sensitive to cAMP, thereby contributing to rapid control of neuronal excitability (21, 31–34). HCN2 is broadly distributed across the CNS—with particularly high expression in the thalamus—activates more slowly, and exhibits greater cAMP sensitivity, making it more susceptible to intracellular signaling pathways (21, 33, 35, 36). By contrast, HCN3 and HCN4 show lower expression levels in the CNS; HCN4 is enriched in regions such as the hypothalamus and olfactory bulb (36, 37). This isoform-specific topography provides the structural basis for precise, region- and process-dependent modulation of neuronal activity by HCN channels.

### 2.2 Electrophysiological functions and physiological roles

The defining electrophysiological feature of HCN channels is hyperpolarization-activated gating: channels open when the membrane potential becomes more negative and conduct the I_h_ current (32, 38). I_h_ is a nonselective cation current that primarily carries Na^+^ influx and K^+^ efflux (38, 39); because Na^+^ entry predominates under physiological conditions, its net effect is to depolarize the membrane from hyperpolarized potentials—a depolarizing rebound (40, 41). On this basis, HCN channels exert a nuanced, bidirectional control over excitability and membrane stability. On the one hand, I_h_-mediated rebound limits excessive hyperpolarization (e.g., following inhibitory postsynaptic potentials, IPSPs), accelerates the return of the membrane potential toward spike threshold, and under certain conditions can lower the excitability threshold (42, 43). On the other hand, channel opening markedly reduces neuronal input resistance, producing a shunt that attenuates synaptic currents and dampens excitatory drive (44). The net impact of I_h_ on excitability thus reflects the balance between these opposing actions and depends critically on channel density, isoform composition, and subcellular distribution (e.g., soma versus dendrites) (45, 46).

HCN channel-mediated I_h_ constitutes an important pacemaker-like depolarizing drive in neurons of rhythm-generating structures such as the thalamus and selected brainstem nuclei, contributing to the setting of resting membrane potential, input resistance, and intrinsic rhythmicity (47–51). In thalamic relay neurons, I_h_ acts in concert with T-type Ca^2+^ channels to determine subthreshold oscillatory properties (52); tuning the strength of I_h_ modulates membrane-potential oscillations and synchrony within the *δ*/*θ* bands, a mechanism tightly linked to thalamo-cortical rhythms across sleep–wake states and to state-dependent modulation of cognition (53). In the hippocampus, HCN channels likewise participate in θ rhythms and the temporal organization of information encoding; HCN1 is particularly enriched in dendrites and constrains spatiotemporal integration by shaping dendritic rectification and shunt properties (44, 54). At the dendritic level, increasing HCN1/I_h_ typically lowers input resistance, accelerates the decay of excitatory postsynaptic potentials (EPSPs), and weakens temporal summation; conversely, downregulating I_h_ prolongs EPSPs and facilitates the propagation of excitatory signals (44). In addition, HCN channels are present at presynaptic terminals and regulate transmitter release via multiple mechanisms: (i) through their Na^+^ permeability, they help set terminal membrane potential and Na^+^ homeostasis, potentially influencing vesicular loading and quantal size (55); and (ii) by coupling to low-threshold Ca^2+^ channels (e.g., Ca_V_3.2), they restrict or tune Ca^2+^ entry, bidirectionally altering the release probability of glutamate or GABA (56, 57). Notably, the impact of HCN channels on presynaptic release is circuit- and terminal-type dependent: in some pathways HCN limits Ca^2+^ influx and constrains transmitter output (56, 57), whereas in others its depolarizing action can promote excitatory transmission (58). Together, these cellular and circuit mechanisms account for the bidirectional influence of HCN/Ih on physiological rhythms and on pathological activity such as epileptiform discharges (44, 49, 54, 59).

## 3 Mechanistic roles of HCN channels in epileptogenesis

### 3.1 Disruption of Ih due to altered HCN channel expression and function

A substantial body of evidence shows that epilepsy is associated with marked changes in HCN channel expression and/or function, resulting in abnormal I_h_—arguably the most immediate mechanism by which HCN channels contribute to epileptogenesis (19, 60). Among these alterations, downregulation of HCN channels is the most frequently observed: within 1 week after status epilepticus (SE; acute phase), dendritic HCN expression in hippocampal CA1 pyramidal neurons begins to decline, accompanied by a hyperpolarizing shift in voltage-dependent activation; this downregulation progresses during the chronic phase of epilepsy (61). By contrast, in chemical models of chronic temporal lobe epilepsy (TLE) induced by kainate or pilocarpine—and in resected tissue from patients with TLE—dentate granule cells often exhibit increased I_h_, which has been associated with elevated HCN1 and/or HCN4 expression, whereas HCN2 is unchanged in most studies; this pattern is commonly interpreted as a compensatory plasticity that may help limit granule-cell excitability (62).

The time course and regional profile of HCN/Ih remodeling are highly context dependent. In CA1 pyramidal neurons, SE triggers a rapid endocytosis and loss of HCN1, followed by decreases in protein and mRNA abundance and a hyperpolarizing shift in voltage-dependent activation—i.e., a gating reconfiguration (16). Mechanistically, reduced I_h_ does not drive the resting membrane potential toward spike threshold; more commonly it renders the membrane slightly more negative. However, because input resistance increases and temporal/spatial summation of EPSPs is enhanced, neurons become more responsive to excitatory inputs, yielding a net facilitation of network excitability. Conversely, augmenting HCN/I_h_ activity increases shunt and reduces dendritic integration, thereby lowering dendritic excitability (45). In epileptic tissue, phosphorylation-dependent downregulation of I_h_ gating constitutes an “acquired channelopathy”; pharmacologic reversal of these phosphorylation changes can restore gating and excitability (63). These adaptations are region- and isoform-specific: CA1 dendrites show the prototypical HCN1 downregulation/I_h_ reduction (16), whereas dentate granule cells more often display I_h_ upregulation (62); cortical changes are more variable and typically of smaller magnitude (64). By contrast, in models of absence epilepsy, I_h_ is often altered in ventrobasal thalamic neurons, and local HCN blockade can reduce or abolish spike–wave discharges (SWD) and absence seizures, underscoring the model-dependent bidirectionality of HCN-mediated effects (65).

### 3.2 Imbalanced interaction between HCN channels and neurotransmitter systems

Glutamate mediates excitatory transmission via AMPA/NMDA receptors, and its excessive activation is closely associated with Ca^2+^ overload, excitotoxicity, and epileptogenesis (66). Presynaptically, HCN channels exert bidirectional regulation on glutamate release: on the one hand, the opening of HCN1 channels in glutamatergic synaptic terminals targeting layer III pyramidal neurons in the mouse entorhinal cortex induces mild and sustained depolarization of the terminals, which inactivates low-threshold T-type Ca^2+^ channels (Ca_V_3.2), reduces Ca^2+^ influx, and inhibits glutamate release; conversely, blocking HCN channels relieves this inhibitory effect and enhances glutamate release (56). On the other hand, the Na^+^ permeability of HCN channels increases the baseline Na^+^ level in terminals, promotes vesicular glutamate loading and quantal size, and thus facilitates glutamate release at certain synapses (e.g., cerebellar/brainstem giant synapses) (55). Postsynaptically, the cAMP/PKA signaling pathway can phosphorylate GluA1 at the Ser845 site, increasing the membrane expression and open probability of AMPA receptors. Several studies have indicated that inhibiting presynaptic HCN1 (accompanied by NMDAR inhibition) can enhance glutamate input and elevate the phosphorylation level/surface expression of GluA1, suggesting an indirect, cross-synaptic coupling between presynaptic HCN channel activity and postsynaptic AMPAR regulation (67).

HCN channels also remodel inhibitory transmission by acting on inhibitory interneurons and presynaptic mechanisms. By changing terminal membrane potential and Ca^2+^ entry, HCN activity can either facilitate or suppress GABA release in a circuit-dependent manner (57, 68). Under epileptic conditions, changes in HCN expression or function are therefore expected to modify the strength and dynamics of inhibitory output and to disturb the balance between excitatory and inhibitory neurotransmitter systems. The consequences of such alterations for neuronal excitability and network stability are further elaborated in Section 3.3.

### 3.3 Abnormal regulation of neuronal excitability and network stability by HCN channels

Epileptic seizures involve not only increased excitability of individual neurons but also abnormal synchronization of neural networks. The I_h_ current mediated by HCN channels participates in the maintenance of network stability by modulating membrane properties (resting membrane potential, input resistance, membrane time constant) and frequency resonance, thereby regulating the rhythmicity and synchronization propensity of neurons and networks (21, 37, 69). In the hippocampal network, HCN channels are particularly critical for the “excitatory-inhibitory (E-I) balance”: CA1 pyramidal neurons highly express HCN1 in their dendrites, and the I_h_ current can accelerate the decay of distal EPSPs, reduce temporal integration, and inhibit distal Ca^2+^ spikes, thereby limiting excessive excitation and synchronization (44, 45, 70). Inhibitory interneurons [e.g., parvalbumin-positive (PV^+^) basket cells] also express HCN/Ih channels; altering the I_h_ current affects their intrinsic excitability and presynaptic release, thereby shaping the inhibitory output onto pyramidal neurons (20, 71). Consistent with this, axon terminals of PV^+^ interneurons in the hippocampal CA1 region highly express HCN1, whose opening can enhance evoked GABA release, whereas pharmacological inhibition or genetic deletion of HCN1 reduces inhibitory output and destabilizes perisomatic inhibition (68). In contrast, in basket cells of layers 5–6 in the prefrontal cortex, HCN channels suppress GABA release by restricting presynaptic T-type Ca^2+^ influx, such that blocking HCN increases the frequency of miniature and spontaneous inhibitory postsynaptic currents (mIPSCs and sIPSCs) (57). These findings indicate that HCN-dependent control of GABA release provides a circuit- and region-specific mechanism for stabilizing or destabilizing inhibitory circuit output, and thus for tuning network stability.

In epilepsy/TLE models, the expression and surface localization of HCN1 in the dendrites of CA1 pyramidal neurons decrease rapidly, and the gating of I_h_ shifts toward more negative potentials (referred to as “acquired dendritic channelopathy”). This leads to increased input resistance, slowed decay of EPSPs, enhanced temporal integration, and ultimately elevated excitability and promoted synchronization of pyramidal neurons (16, 46). At the presynaptic level, HCN channels control presynaptic transmitter output/synaptic efficacy in a pathway-specific manner: in some inhibitory synapses, HCN activity limits GABA release via T-type Ca^2+^ channel coupling (57); whereas in the calyx of Held (a giant glutamatergic synapse) HCN-dependent Na^+^ influx increases quantal size and vesicular glutamate loading (55). Abnormal synchronization of firing and epileptogenicity can be increased not only in the hippocampus but also in cortical networks. For example, mice with selective reduction of HCN1 exhibit elevated cortical excitability and increased epileptogenicity, indicating a protective role of HCN1 (46). Taken together, HCN channels maintain E-I balance by simultaneously regulating the intrinsic excitability and presynaptic release of both pyramidal neurons and inhibitory interneurons. When HCN function undergoes spatiotemporally specific downregulation or mismatch, neural networks become more prone to abnormal synchronous firing, thereby triggering epilepsy (16, 21, 37, 69, 72).

## 4 Subtype-specific alterations of HCN channels in epilepsy

### 4.1 Temporal lobe epilepsy

TLE is the most common type of focal epilepsy in adults, accounting for a major proportion of cases undergoing surgical treatment. Its most prevalent pathological subtype is hippocampal sclerosis (HS). Recent methodological and review work has reinforced HS as the most common histopathological abnormality in drug-resistant TLE and has emphasized cohort-dependent variability in its reported frequency (73–75). Studies of resected human tissue and acquired epilepsy models demonstrate region-specific HCN remodeling in TLE. In the hippocampal CA1 region in MTLE-HS, HCN1 is commonly reduced at the transcript and/or protein level, with evidence for diminished dendritic expression; in acquired post-status epilepticus models, this loss of dendritic HCN1/I_h_ is accompanied by a hyperpolarizing shift in I_h_ voltage dependence, consistent with an “acquired dendritic h-channelopathy,” which can increase CA1 pyramidal-cell responsiveness by elevating input resistance and strengthening dendritic integration (16, 76, 77). In contrast, in dentate gyrus (DG) granule cells, multiple studies on human and rodent models of chronic TLE have shown that I_h_ density is commonly upregulated, consistent with increased expression of HCN1/HCN4 (HCN2 expression remains unchanged in most cases). This alteration is considered compensatory, as it helps restrict excessive excitation and spatiotemporal integration of DG (62, 78).

In chemical epileptogenic models, the time-course changes of HCN1 have been characterized: Rapid endocytosis and surface loss of dendritic HCN1 occur within hours to days after SE, followed by a decrease in protein levels and mRNA expression (16). Notably, these changes are particularly prominent in the hippocampal CA1 region and exhibit distinct regional differences along the dorsoventral axis, with more pronounced alterations in the dorsal CA1 compared to the ventral subdivision (77). Taken together, HCN remodeling in TLE is dependent on “brain region-subtype-time phase”: CA1 is dominated by HCN1 downregulation/I_h_ reduction (promoting excitation and epileptogenesis), while DG more frequently exhibits HCN/Ih upregulation (tendency toward compensation). This bidirectional plasticity, together with impaired network homeostasis, may contribute to epileptogenesis and the persistence of chronic epileptic networks (16, 75–78).

### 4.2 HCN channel genetic variants and epilepsy

#### 4.2.1 HCN1 genetic variants and epilepsy

HCN1 is one of the key molecules mediating I_h_ in the central nervous system. *De novo* missense variants in *HCN1* were first reported in 2014 as a cause of early infantile epileptic encephalopathy (EIEE)/developmental and epileptic encephalopathy (DEE), characterized by infantile onset, frequent drug resistance, and fever/heat sensitivity (19). Subsequent cohort studies have further expanded the variant spectrum and phenotypic heterogeneity, covering Dravet-like epilepsy and various types of refractory epilepsy in infants/children, and suggesting differences in drug responses (10). Functionally, *HCN1* variants can exhibit bidirectional mechanisms: both gain-of-function (GOF) effects (e.g., causing voltage-dependent shifts, accelerated activation, and increased leak currents) and loss-of-function (LOF) effects (e.g., decreased current density), leading to imbalance in network excitation and rhythm (79). Corresponding knock-in mouse models directly confirm the pathogenicity of these variants and abnormal drug responses, providing a model basis for intervention strategies (80, 81). At the translational level, Org-34167, a brain-penetrant broad-spectrum HCN modulator, was recently shown in a study published in Epilepsia to correct the voltage dependence and leak currents of various *HCN1*-DEE variant channels. Additionally, it reduced hyperexcitability and improved behavioral phenotypes in *Hcn1*^M249L^ mice, suggesting the feasibility of mechanism-targeted small molecules (though side effects such as tremors need to be balanced) (82). To date, peer-reviewed reports demonstrating robust seizure/behavioral rescue in HCN1-DEE models via HCN1 gene replacement or genome editing remain limited (69).

#### 4.2.2 HCN2 genetic variants and epilepsy

HCN2 channels are among the key HCN channels in the brain. Studies have demonstrated that abnormal *HCN2* function is closely associated with epilepsy, especially absence epilepsy (83). First, in Hcn2 knockout (or mutant) animal models, the I_h_ current in thalamic relay neurons is nearly abolished, and the resting membrane potential is significantly hyperpolarized. This renders neurons more prone to entering a burst oscillation state, ultimately inducing typical ~5 Hz SWD and absence-like seizures (84). Second, human genetic studies have identified rare variants of *HCN2* in multiple epilepsy patients, involving two types of mechanisms: GOF and LOF (85, 86). The bidirectional mechanisms of these variants indicate that either a decrease or an increase in *HCN2* function alone may disrupt thalamocortical rhythms and trigger epilepsy. Finally, mechanistic studies suggest that in the thalamocortical circuit, the regulation of I_h_ must be maintained in a “moderate state”: both excessively low I_h_ (e.g., HCN2 deficiency) and excessively high I_h_ (e.g., HCN upregulation in certain models) may disrupt rhythmic homeostasis (37). Therefore, *HCN2*-related epileptogenesis exhibits activity-level (dosage) dependence, brain-region/circuit dependence, and mechanism dependence. Precise regulation of HCN2 channels or individualized interventions targeting mutant forms (such as channel modulators or gene therapy) holds potential clinical significance.

#### 4.2.3 HCN3 genetic variants and epilepsy

The role of *HCN3* in epileptogenesis has not been fully investigated to date. One study recruited 298 epilepsy patients, and through Sanger sequencing, three rare heterozygous variants (R457H, P630L, R661Q) in the *HCN3* gene were identified. *In vitro* functional analyses showed that the R457H and R661Q variants significantly reduced cellular current density, which may affect neuronal excitability, while the P630L variant had no effect on ion channel current. Structural analysis indicated that the R457H and R661Q variants decreased the stability of HCN3 channels. Epilepsy was well-controlled after treatment in patients carrying these variants. This study suggests that *HCN3* may be a novel candidate gene in the pathogenesis of epilepsy, but additional cases, segregation evidence, and *in vivo* validation are required (87). In addition, a systematic review summarizing published HCN channelopathy cases reported two postmortem cases carrying rare *HCN3* variants (K69R and P630L) that were classified as unclassified/unknown epileptic syndromes and died of SUDEP, raising the possibility of a link between *HCN3* variation and SUDEP risk; however, evidence remains extremely limited and requires confirmation in large, well-phenotyped cohorts (37).

#### 4.2.4 HCN4 genetic variants and epilepsy

HCN4 channels play a crucial role in regulating neuronal excitability, rhythmic firing, and synaptic transmission (21, 88, 89), and have been implicated as regulators of epilepsy susceptibility and network-level excitability (90). To date, studies have reported *HCN4* variants and abnormal expression implicated in epilepsy subtypes, including idiopathic generalized epilepsy and mTOR-related epileptic syndromes (37, 91–94). In animal models, relevant studies have further support a role for abnormal HCN4 expression in epileptogenesis (88, 95, 96), indicating that HCN4 channels play a significant role in epilepsy and may represent a potential therapeutic target (88).

### 4.3 Drug-resistant epilepsy

A growing body of evidence from resected human tissue and animal models suggests that acquired abnormalities of HCN channels are associated with drug-resistant focal epilepsies, particularly MTLE-HS. In surgical specimens from human MTLE-HS, HCN1 (and, in some studies, HCN2) mRNA and protein levels in the hippocampus are reduced; subcellular localization analyses further indicate diminished dendritic membrane expression, consistent with decreased dendritic I_h_ availability/function (76). At the mechanistic level, epileptiform activity or SE can induce rapid endocytosis/mislocalization and downregulated gating of dendritic HCN1 (16, 63), leading to increased dendritic input resistance, abnormal oscillatory activity and temporal integration, thereby promoting network hyperexcitability (referred to as “acquired channelopathy”) (46, 72). This plasticity is finely regulated by channel chaperones and cytoskeletal proteins: Different splice variants of TRIP8b bidirectionally regulate the surface localization and gating of HCN1 (97, 98), while Filamin-A promotes dynamin-dependent endocytosis of HCN1 and restricts I_h_ (99), suggesting that targeting the trafficking pathway may be a strategy to restore dendritic HCN1. Furthermore, in models of prolonged febrile seizures during development and some acquired epilepsy models, an increased proportion of HCN1/HCN2 heterotetramers and a decreased HCN1/HCN2 ratio have been observed, which alters the kinetics and cAMP sensitivity of I_h_. This “subunit reassembly” is thought to contribute to the development of sustained hyperexcitability (100).

In parallel, increased expression and overactivity of the efflux transporter P-glycoprotein (P-gp/ABCB1) has been reported in drug-resistant TLE/MTLE-HS, including upregulation within epileptogenic regions and at the blood–brain barrier (101). PET studies using P-gp substrate tracers further suggest regional P-gp overactivity in pharmacoresistant TLE, supporting an association with the “transporter hypothesis,” whereby enhanced efflux may reduce target-site exposure to some ASMs (102). Taken together, the acquired downregulation/mislocalization of HCN channels (rendering neurons more excitable) and enhanced blood–brain barrier (BBB) efflux (reducing drug exposure) may concurrently drive reduced treatment efficacy in DRE. Future interventions could focus on restoring the expression and localization of dendritic HCN1 (e.g., targeting the TRIP8b/Filamin-A axis), optimizing HCN-targeted modulators, and combining these approaches with transporter modulation strategies.

## 5 Progress in HCN channel-based epilepsy therapy

### 5.1 HCN channel agonists

Although no selective HCN channel openers/activators have entered routine clinical practice to date, some marketed ASMs have been reported to enhance the I_h_ current indirectly or in a context-dependent manner. For example, lamotrigine increases Ih in human layer 2/3 neocortical pyramidal neurons from pharmacoresistant epilepsy patients, decreases input resistance, and reduces EPSP amplitude and temporal summation; the biophysical basis of I_h_ enhancement may vary across preparations (103). Gabapentin has also been shown to increase I_h_ amplitude in rat CA1 pyramidal neurons mainly by increasing conductance without significant changes in Ih activation properties or kinetics, and this effect has been proposed to contribute to its antiseizure action (104). In addition, pharmacological elevation of cAMP (e.g., forskolin) can suppress PTZ-induced seizures in rodents; because cAMP facilitates HCN channel opening, these findings are consistent with (but do not by themselves prove) an antiseizure potential of the cAMP–HCN–Ih pathway (105, 106).

However, recent channelopathy studies suggest that “activating HCN channels” is not universally beneficial. Some GOF mutations in HCN1 channels (e.g., M305L) enhance I_h_ and cause severe epileptic seizures (81); in such patients, even lamotrigine-like drugs may exacerbate symptoms. This indicates that if HCN channel openers are to be developed in the future, strict differentiation should be made based on channel subtypes, brain regions, and mutation backgrounds to achieve precise regulation. Recent molecular biology studies have also explored “targeted modulation” of HCN1 channels using nanobodies, which partially corrected abnormal voltage dependence *in vitro* (107), providing a proof-of-concept/biophysical basis for “selective HCN modulators.”

### 5.2 HCN channel inhibitors

HCN channel inhibitors regulate neuronal rhythmic activity and excitability by suppressing the I_h_ current, but their role in epilepsy is not unidirectional. Early studies suggested that such inhibitors might enhance neuronal excitability, thereby inducing or exacerbating epileptic seizures. This view was partially validated in the Genetic Absence Epilepsy Rats from Strasbourg (GAERS) model: intracerebroventricular injection of a high dose (7 μg) of ZD7288 increased the number of SWD on EEG, indicating that HCN channel inhibition may promote epileptiform activity under certain dose- and time-dependent conditions (108). It should be noted, however, that ZD7288 is not a fully selective HCN blocker and can influence synaptic transmission through off-target actions, which may contribute to its bidirectional effects. In contrast, local application of the Ih blocker ZD7288 within the thalamic ventrobasal (VB) nucleus has been shown to suppress SWD-locked burst firing of VB relay neurons at higher concentrations (2 mM), while no consistent effect on SWD components recorded on the epidural EEG was observed, highlighting the region-specific nature of I_h_ modulation (109); in TLE models, selective inhibition of HCN channels in the subiculum-anteroventral thalamic nucleus (AV) circuit suppressed epilepsy generalization, demonstrating that its effects depend on differences at the circuit level (108, 110).

Furthermore, HCN channel inhibitors may also exhibit antiepileptic potential in certain types of epilepsy. EC18, a representative HCN4-preferring blocker, significantly reduced network excitability and epileptogenicity in a PTZ-induced convulsion model in wild-type mice, but had no effect in *Hcn4* conditional knockout mice—this strongly supports its antiepileptic mechanism via targeting HCN4 channels (88). Another brain-penetrant broad-spectrum HCN channel modulator, Org-34167, has also been shown to modulate neural network excitability and exhibit antiepileptic potential (82); however, high doses can induce motor coordination deficits, suggesting that dose optimization and safety evaluation remain necessary (111). Notably, gabapentin, a traditional anticonvulsant, can also modulate the voltage dependence and kinetic properties of HCN4 channels—this effect may be involved in its antiepileptic mechanism, though direct causal verification is currently lacking (112).

### 5.3 Other HCN channel-related therapeutic strategies

Under the mechanism-based stratification of DRE, in addition to direct “activation/inhibition” of HCN channels, the I_h_ current and network synchronization can also be indirectly corrected through multiple levels, including neural networks, co-channels, second messengers, trafficking/endocytosis, and neural circuits: (1) Network neuromodulation—Deep brain stimulation of the anterior nucleus of the thalamus (ANT-DBS) has been shown to reduce seizure frequency in the long term in real-world and follow-up studies (113, 114). Its mechanism is more likely to involve decoupling the limbic-thalamocortical network, thereby potentially affecting HCN/I_h_ (though direct evidence in humans is lacking for direct upregulation of any specific HCN subtype); (2) Co-channel compensation—Enhancing the dendritic M-current (mediated by Kv7/KCNQ channels) can work with HCN channels to produce a “shunting/damping” effect, reducing dendritic integration and inhibiting abnormal synchronization. The next-generation Kv7 opener XEN1101 demonstrated significant seizure reduction in a Phase IIb randomized controlled trial for adult focal epilepsy and is being evaluated in Phase III (44, 115); (3) Second messenger fine-tuning—The neuromodulator-cAMP axis can induce a positive shift in HCN (particularly HCN2/4) gating. Imaging and mechanistic evidence linking vagus nerve stimulation (VNS) to the locus coeruleus-norepinephrine system provides indirect clinical support for “modulating HCN via cAMP” (21, 116); (4) Trafficking/endocytosis targets—Targeting the TRIP8b-HCN interaction can simultaneously regulate membrane expression and gating (proof-of-concept has been achieved with NUCC-0200590, a small molecule with demonstrated neuronal effects as a proof-of-concept tool) (117, 118); inhibiting Filamin-A-mediated endocytosis of HCN1 offers another approach to “correct acquired channelopathy”; (5) Circuit targeting—In mice, HCN1 expressed in the projection from the subiculum to the ANT promotes the generalization of hippocampal seizures to the thalamocortex. Downregulating or blocking HCN1 in this pathway can alleviate seizures, providing a potential “molecular-circuit” basis for site selection and parameter optimization of DBS/responsive neurostimulation (RNS) (110).

## 6 Conclusion

HCN channels are key regulators of neuronal excitability and network stability and play important roles in the pathogenesis of epilepsy. In epileptic states, abnormal expression, dysfunction, or gene mutations of HCN channels can induce or exacerbate seizures via I_h_ dysregulation, disruption of neurotransmitter balance, distortion of E-I balance, and impaired network synchrony. HCN-targeted strategies, including pharmacological modulators, gene-based therapies, and neuromodulation, have shown promising antiepileptic effects in animal models, and some have progressed into early preclinical evaluation.

However, the intrinsic bidirectionality of HCN function poses a major challenge for therapeutic translation. Increasing or decreasing I_h_ can produce opposite effects: I_h_ supports both depolarizing rebound and shunting inhibition; presynaptic HCN channels bidirectionally regulate glutamate and GABA release in a circuit- and terminal type-dependent manner; and HCN remodeling and genetic variants include both GOF and LOF changes that are often epileptogenic. *In vivo*, the same non-selective HCN modulator may suppress seizures in one model or brain region but aggravate them in another. Thus, simple “HCN openers” or “HCN blockers” are unlikely to be universally beneficial and may even worsen seizures in patients whose predominant pathology involves the opposite direction of functional change.

Future work should therefore focus on three directions. First, the development of subtype- and state-selective HCN modulators, guided by structural biology and in silico design, is needed to overcome the limitations of non-specific agents such as ZD7288. Second, CRISPR-based editing and other gene-targeted approaches for HCN channelopathies should be explored, with careful functional stratification of variants and optimization of viral delivery to epileptogenic circuits. Third, integrating multi-omics profiling and long-read sequencing into clinical cohorts may enable robust stratification of epilepsy subtypes and support personalized HCN-targeted interventions for drug-resistant patients.

With the innovative development and deep application of key technologies such as single-cell genomics and precision gene editing in HCN channel research, our understanding of the mechanisms related to HCN channels in epilepsy will continue to deepen. This is expected to overcome translational bottlenecks and provide novel targeted targets for the precise diagnosis and treatment of epilepsy, especially drug-resistant epilepsy.
